# RFRP neurons are critical gatekeepers for the photoperiodic control of reproduction

**DOI:** 10.3389/fendo.2012.00168

**Published:** 2012-12-18

**Authors:** Valérie Simonneaux, Caroline Ancel

**Affiliations:** Neurobiologie des Rythmes, Institut des Neurosciences Cellulaires et Intégratives, UPR CNRS 3212Strasbourg, France

**Keywords:** RFamide peptide, RFRP-3, GPR147, kisspeptin, seasonal reproduction, melatonin

## Abstract

Seasonally breeding mammals rely on the photoperiodic signal to restrict their fertility to a certain time of the year. The photoperiodic information is translated in the brain via the pineal hormone melatonin, and it is now well-established that it is the variation in the duration of the nocturnal peak of melatonin which synchronizes reproduction with the seasons. The Syrian hamster is a long day breeder, and sexual activity is therefore promoted by exposure to a long day photoperiod and inhibited by exposure to a short day photoperiod. Interestingly, in this species electrolytic lesion of the mediobasal hypothalamus abolishes the short day-induced gonadal regression. We have shown that the expression of a recently discovered neuronal population, namely *RFamide-related peptide* (*rfrp*) neurons, present in the mediobasal hypothalamus, is strongly down-regulated by melatonin in short day conditions, but not altered by circulating levels of sex steroids. The role of *rfrp* and its product RFRP-3 in the regulation of reproductive activity has been extensively studied in mammals, and our recent findings indicate that this peptide is a potent stimulator of the reproductive axis in the Syrian hamster. It induces a marked increase in GnRH neuron activity and gonadotropin secretion, and it is able to rescue reproductive activity in short day sexually inactive hamsters. Little is known about the localization of the RFRP-3 receptor, GPR147, in the rodent brain. Accumulating evidence suggests that RFRP-3 could be acting via two intermediates, the *GnRH* neurons in the preoptic area and the *Kiss1* neurons in the arcuate nucleus, but future studies should aim at describing the localization of *Gpr147* in the Syrian hamster brain. Altogether our data indicate that the *rfrp* neuronal population within the mediobasal hypothalamus might be a serious candidate in mediating the photoperiodic effects of melatonin on the regulation of the reproductive axis.

## MAMMALS USE THE RHYTHMIC SECRETION OF THE PINEAL HORMONE MELATONIN TO SYNCHRONIZE REPRODUCTION WITH THE SEASONS

A large number of species restrict their fertility to a particular time of the year to ensure that the birth of the offspring occurs during the most favorable season. To determine the time of the year and synchronize their reproductive activity accordingly, mammals use the highly reproducible annual variations in light duration (or photoperiod). Photoperiod is transduced by a photoneuroendocrine system composed of the retina, the suprachiasmatic nucleus (seat of the master circadian clock) and the pineal gland which releases the hormone melatonin exclusively at night, so that the duration of the secretion varies according to night length. Therefore, the photoperiodic variations in circulating levels of melatonin throughout the year provide the body with a robust and reproducible representation of the seasons (Simonneaux and Ribelayga, 2003). It has long been established that photoperiodic variations in the duration of the nocturnal peak of melatonin synchronize reproduction in seasonal species like sheep or hamsters (Hoffman and Reiter, 1965; Carter and Goldman, 1983; Pevet, 1988; Bartness et al., 1993; Malpaux et al., 2001).

Syrian hamsters are long day breeders, meaning that they are sexually active in long day (LD: 14 light/10 h dark) conditions. Upon exposure to short day (SD: 10 light/14 h dark) conditions, they undergo a dramatic inhibition of reproductive activity within 8–10 weeks manifested by a marked atrophy of the gonads and accessory organs resulting in low levels of circulating sex steroids. Surgical removal of the pineal gland before exposure to SD conditions prevents hamsters from undergoing sexual inactivation. Conversely, exogenous melatonin injections mimicking SD conditions in hamsters raised in LD conditions induce sexual inactivation. In contrast to small rodents, large mammals with a longer gestation time like sheep are SD-breeders; they are sexually active in SD conditions and become quiescent after transfer to LD conditions. Although the reproductive timing is opposite in hamsters and sheep, in both cases the circulating levels of melatonin synchronize reproduction with photoperiod. However, why the reproductive systems of LD- and SD-breeders respond in opposite ways to the same melatonin signal is not known.

## MELATONIN ACTS ON THE PARS TUBERALIS TO TRANSMIT PHOTOPERIODIC INFORMATION

It is clear that melatonin does not act directly on GnRH neurons and responsiveness to GnRH does not change with photoperiod (Urbanski et al., 1991). Melatonin binding sites are found in a number of brain structures but with considerable species differences (Masson-Pevet et al., 1994). Besides, a high density of melatonin receptors has been identified in the pars tuberalis of the adenohypophysis in a large number of mammalian species (Masson-Pevet and Gauer, 1994). Notably, the pars tuberalis cells expressing melatonin receptors synthesize thyroid-stimulating hormone (TSH) in a photoperiod/melatonin-dependent manner, with a higher level of expression in LD conditions (Klosen et al., 2002; Dardente et al., 2003, 2010). TSH produced by the pars tuberalis has been recently recognized as a key messenger through which melatonin acts on the gonadotropic axis for the seasonal control of reproduction. TSH acts on a specialized glial cell type of the hypothalamic ependymal wall, the tanycytes, to induce a marked up-regulation of the thyroid hormone-activating enzyme deiodinase 2 (Dio2) which in turn increases local concentrations of the bioactive T3 thyroid hormone (Yoshimura et al., 2003; Hanon et al., 2008, 2010; Nakao et al., 2008). In quail (Yoshimura et al., 2003) and Siberian hamsters (Barrett et al., 2007) local T3 administration was reported to increase reproductive activity although through unknown mechanisms.

Altogether these observations point to the pars tuberalis as a key site for the integration of the endocrine melatoninergic message for the seasonal regulation of reproductive activity. However, the central reproductive site(s) actually controlled by the melatonin/TSH system is(are) still unknown. Various structures in the mediobasal hypothalamus have been proposed to be direct or indirect sites of action for melatonin, particularly in the sheep (Malpaux et al., 1998) and hamster (Maywood and Hastings, 1995).

## RFRP-3 NEURONS LOCATED IN THE MEDIOBASAL HYPOTHALAMUS ARE STRONGLY REGULATED BY MELATONIN

In the Syrian hamster, the dorsal part of the mediobasal hypothalamus appears as a key structure for the photoperiodic regulation of reproductive activity since it contains melatonin binding sites and its ablation by electrolytic lesion prevents the inhibitory effect of melatonin on reproductive activity (Maywood et al., 1996). Recently, we reported that neurons located in this hypothalamic area express the *RFamide-related peptide* (*rfrp)* gene in a photoperiodic-dependent manner in Siberian and Syrian hamsters (Revel et al., 2008).

The *rfrp* gene was discovered in 2000 in mammals (Hinuma et al., 2000), concurrently with the discovery of its avian ortholog *gonadotropin-inhibitory hormone* (*gnih*; Tsutsui et al., 2000). The *rfrp* and *gnih* genes were found to produce new peptides of the RFamide family of peptides, which share a common C-terminal LPXRFamide (X = L or Q) motif. In the quail, GnIH was shown to act directly at the level of the pituitary to inhibit gonadotropin release (Tsutsui et al., 2000). In mammals, the *rfrp* gene is expressed in neurons located in the mediobasal hypothalamus and encodes a precursor that produces two peptides, RFRP-1 and RFRP-3 (Ukena and Tsutsui, 2001; Kriegsfeld et al., 2006; Clarke et al., 2008; Dardente et al., 2008; Revel et al., 2008; Smith et al., 2008; Rizwan et al., 2009). The demonstration that GnIH is a potent inhibitor of gonadotropin release in birds spurred great interest in the roles of RFRP-1 and particularly RFRP-3 in the regulation of endocrine functions in mammals (Bentley et al., 2010; Kriegsfeld et al., 2010; Smith and Clarke, 2010; Tsutsui et al., 2010 for reviews).

**FIGURE 1:**
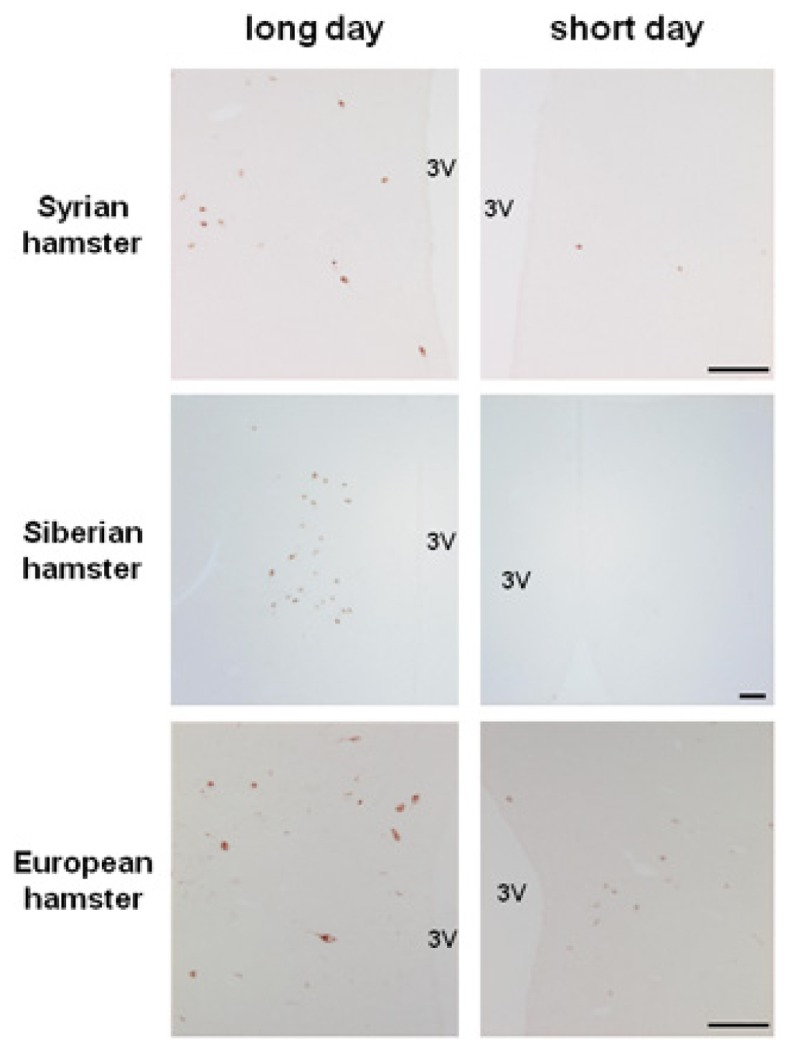
**Photoperiodic variations in RFRP immunoreactivity in the dorsomedial hypothalamus of male Syrian, Siberian, and European hamsters raised in long day or short day conditions.** Scale bar = 100 μM, 3V: third ventricle. RFRP antibody was generously provided by Dr. Greg Anderson; Syrian and Siberian hamster pictures were kindly provided by Julien Bartzen and European hamster pictures by Cristina Saenz de Miera.

In Syrian and Siberian hamsters, we observed that the level of *rfrp* mRNA is strongly down-regulated in sexually inactive SD-adapted animals (Revel et al., 2008). This variation is solely photoperiodic as there are no daily changes in *rfrp* mRNA levels either in LD or SD conditions. In both species, the SD-induced decrease in *rfrp* gene expression is associated with a similar decrease in peptide immunoreactivity in perikarya and fibers (Revel et al., 2008; Mason et al., 2010; Ubuka et al., 2012; **Figure 1**). We have recently found a similar SD-induced inhibition of *rfrp* expression in other LD-breeders, notably the European hamster (**Figure 1**) and the jerboa (Janati et al., 2012). Strikingly, in sheep, a SD-breeder, two studies reported that hypothalamic *rfrp* mRNA levels and RFRP immunoreactivity are also reduced in SD conditions while animals are sexually active (Dardente et al., 2008; Smith et al., 2008). In contrast, in the non-photoperiodic rat *rfrp* mRNA levels are not modified by photoperiodic conditions (Revel et al., 2008).

In the Syrian hamster, we demonstrated that the SD down-regulation of *rfrp* expression is not due to the lower levels of circulating sex steroids since neither testosterone implants in sexually inactive SD hamsters, nor testis ablation in LD-adapted hamsters altered the levels of *rfrp* mRNA. This lack of major sex steroid feedback on *rfrp* expression is in agreement with other studies conducted in rats, mice, Siberian hamsters, and sheep (Smith et al., 2008; Quennell et al., 2010; Poling et al., 2012; Ubuka et al., 2012). Of note however, studies in female Syrian hamsters reported that RFRP neurons contain Er-α and respond to estrogen administration (Kriegsfeld et al., 2006; Gibson et al., 2008) and RFRP expression in ewe is reduced during the preovulatory period (Clarke et al., 2012).

Importantly, we found that pineal gland ablation before transferring hamsters to SD conditions, a protocol which prevents the SD-induced inhibition of reproductive activity, prevented the decrease in *rfrp* mRNA levels (Revel et al., 2008). Conversely, repeated melatonin injections during the late afternoon to LD-adapted hamsters, a protocol known to inhibit reproductive activity, also induced a marked decreased in *rfrp* mRNA levels (Revel et al., 2008). In the Siberian hamster as well, the down-regulation of *rfrp* expression in SD conditions is induced by melatonin (Ubuka et al., 2012). Remarkably, in the quail, melatonin also regulates GnIH expression but in an opposite manner compared to mammals. Melatonin binds to Mel1c receptors located on GnIH neurons to increase GnIH synthesis and release, and as a consequence, expression of this inhibitory peptide is increased under SD conditions (Ubuka et al., 2005).

Altogether these observations indicate that in a number of seasonal mammalian species, *rfrp* expression is decreased in SD conditions regardless of whether the species is a LD- or a SD-breeder. Experiments with melatonin manipulation carried out in Syrian and Siberian hamsters demonstrate that *rfrp* down-regulation results from the larger production of melatonin in SD conditions. It is tempting to speculate that melatonin may act primarily on RFRP neurons in the mediobasal hypothalamus to control seasonal reproduction. Several observations, however, indicate that this central effect of melatonin is probably indirect. Melatonin binding sites are found in the area where RFRP neurons are located in the Syrian hamster, but this is not the case in the other seasonal species like the Siberian and European hamsters or the sheep. Furthermore, in the Syrian hamster, we found that at least 3 weeks of daily melatonin administration is required to induce a significant reduction in the level of *rfrp* mRNA (Revel et al., 2008) whereas the pineal hormone is much faster to control the expression of other photoperiodically regulated genes like *tsh* in the pars tuberalis or *deiodinase 2* in the tanycytes (Revel et al., 2006a; Yasuo et al., 2007; Dardente, 2012). Therefore, it appears likely that there is an intermediate between the endocrine melatoninergic message and the photoperiodic regulation of RFRP expression, and it might be interesting to investigate whether it is the melatonin-driven TSH/T3 signal.

## RFRP-3 STIMULATES THE GONADOTROPIC AXIS AND RESCUES REPRODUCTIVE ACTIVITY IN PHOTO-INHIBITED HAMSTERS

An increasing number of studies now indicate that RFRP-3 is implicated in the regulation of mammalian reproductive function (Bentley et al., 2010; Tsutsui et al., 2010 for reviews). In mice RFRP-3 was found to exhibit rapid and repeatable inhibitory effects on the firing rate of a subpopulation of GnRH neurons (Ducret et al., 2009). In male rats, intracerebroventricular (icv) RFRP-3 significantly suppresses all facets of sex behavior and also significantly reduces plasma levels of luteinizing hormone (LH; Johnson et al., 2007; Pineda et al., 2010). In female rats, chronic icv infusion of RFRP-3 causes a dose-dependent inhibition of GnRH neuronal activation at the LH surge peak and also suppresses neuronal activation in the anteroventral periventricular region, which provides stimulatory input to GnRH neurons (Anderson et al., 2009). In ovariectomized mature rats, intravenous administration of RFRP-3 significantly reduces plasma LH concentrations (Murakami et al., 2008). Finally, in the ovine and bovine species, RFRP-3 administration inhibits gonadotropin release (Clarke et al., 2008; Kadokawa et al., 2009; Sari et al., 2009) although this is still controversial (Caraty et al., 2012).

**FIGURE 2:**
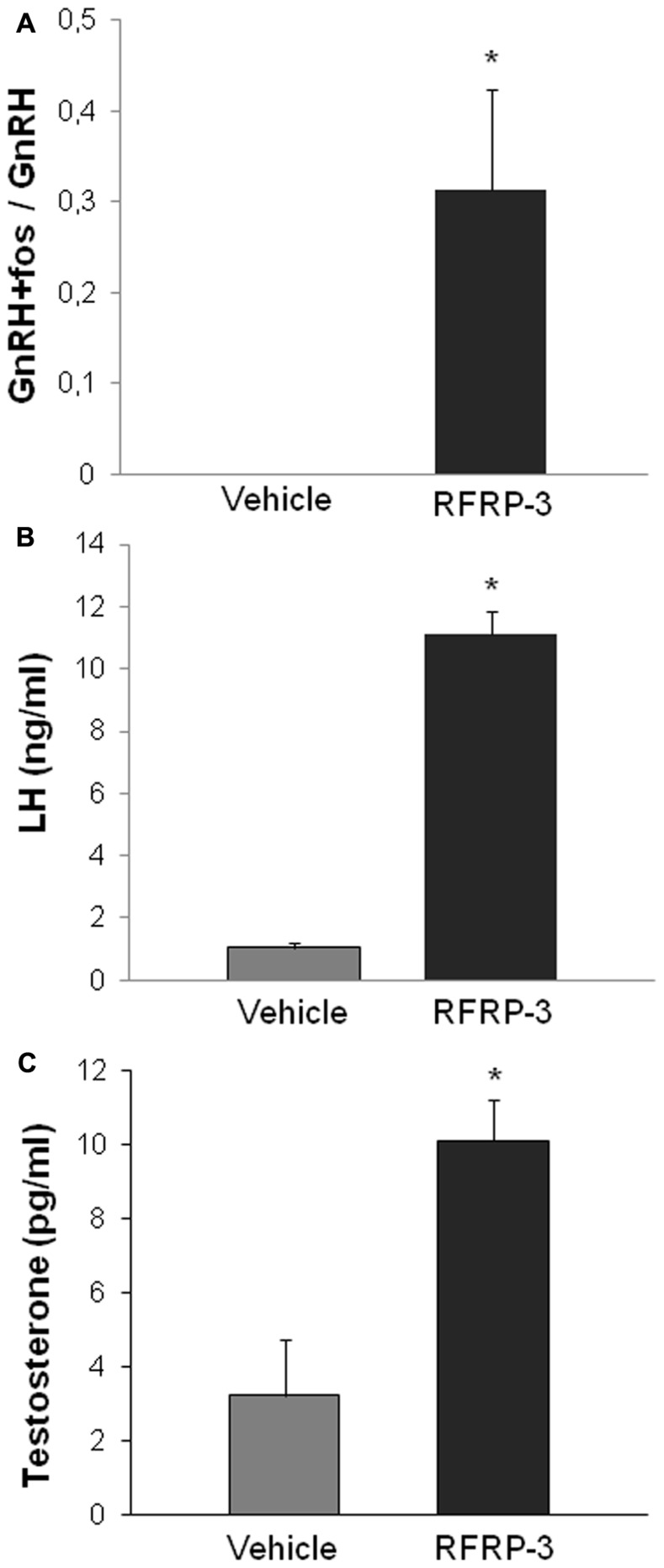
**Acute intracerebroventricular administration of RFRP-3 (1500 ng) activates c-FOS expression in GnRH neurons (A) and increases LH production (B) and circulating levels of testosterone (C) in sexually active long day-adapted male Syrian hamsters.** *p < 0.05 when compared to vehicle-treated animals. Adapted from Ancel et al. (2012).

**FIGURE 3:**
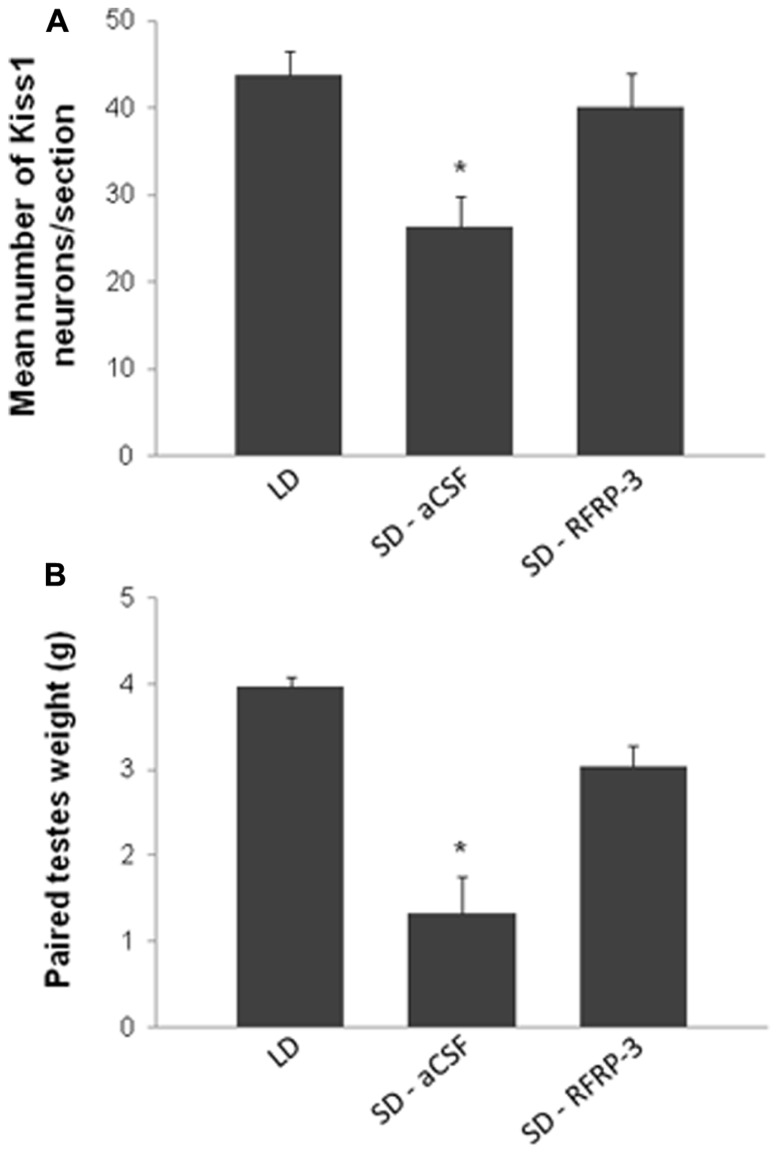
**Chronic intracerebroventricular administration of RFRP-3 [12 μg/day for 5 weeks in artificial cerebrospinal fluid (aCSF)] in sexually inactive short day (SD)-adapted male Syrian hamsters increases kisspeptin expression in the arcuate nucleus (A) and testicular weight (B) up to values comparable to animals kept in long day (LD) conditions.** *p < 0.05 when compared to LD or RFRP-3 treated SD animals. Adapted from Ancel et al. (2012).

Until recently, and based on the plethora of publications supporting this hypothesis, it was assumed that RFRP-3 functioned in mammals as GnIH functioned in birds and served as an inhibitory component regulating the hypothalamic–pituitary–gonadal axis. However, this statement was somehow contradictory with our observation of an increased synthesis of RFRP in LD-adapted sexually active hamsters. We have recently reported novel findings in the male Syrian hamster (Ancel et al., 2012) which have led to call this assumption into question, concurrently with another group working on the male Siberian hamster (Ubuka et al., 2012). In the male Syrian hamster, we reported that acute icv administration of RFRP-3 stimulates GnRH cell activity, gonadotropin release, and testosterone production under LD conditions (**Figure 2**; Ancel et al., 2012). In the same manner, in SD-adapted male Syrian hamsters a single central injection of RFRP-3 increases gonadotropin release [LH (ng/ml) vehicle 1.72 ± 0.31 vs. RFRP-3 4.36 ± 0.82; *n* = 6; *p* < 0.05]. Furthermore, under the same photo-inhibitory conditions, 5 weeks of continuous central administration of RFRP-3 to male Syrian hamsters produces a complete reactivation of the reproductive axis, manifested by increased testis weight and circulating levels of testosterone, similar to those observed in LD conditions (**Figure 3A**; Ancel et al., 2012). In the Siberian hamster, while administration of RFRP-3 in LD conditions inhibits gonadotropin release, the same protocol stimulates gonadotropin secretion in SD conditions (Ubuka et al., 2012). Remarkably, these findings of a stimulatory action of RFRP-3 on the male hamster reproductive axis are in sharp contrast with a previous study reporting an inhibitory effect of icv GnIH in ovariectomized female Syrian hamsters (Kriegsfeld et al., 2006). In LD conditions, reproductive activity of female rodents displays a well-described estrous cycle, characterized by varying levels of circulating gonadotropins and sex steroids. It has been hypothesized that RFRP-3 might be an inhibitory component of the negative feedback loop which regulates the estrous cycle, since RFRP cellular activity is decreased at the time of the LH surge in the Syrian hamster (Gibson et al., 2008). In this context it would be interesting to determine whether the effect of RFRP-3 on the female reproductive axis depends on the stage of the estrous cycle at which it is administered.

Taken together, these data indicate that there are certainly species- and gender-dependent differences in the involvement of RFRP-3 in the regulation of reproductive activity. As a consequence one might be cautious when calling the mammalian peptide “GnIH” based on its effect in birds, and in the light of the recent reports on work carried out in male hamsters the peptide should be termed “RFRP-3.” From a seasonal point of view, when considering LD- (hamster) and SD- (sheep) breeders, it is remarkable to find that while the expression of the peptide in increased in LD conditions in both kinds of breeders, the effect of the peptide on the gonadotropic axis is opposite, as it is stimulatory in hamsters and inhibitory in sheep. This suggests a non-conserved role and/or site of action for RFRP-3 across seasonally breeding species. It is tempting to speculate that RFRP neurons may play a key role in discriminating between long and short day breeders because RFRP expression is down-regulated by a short day profile of melatonin in both kinds of seasonal breeders but the peptide appears to have an opposite effect on the reproductive axis, being stimulatory in long day and inhibitory in short day breeders. Complementary experiments will have to be carried out in other species to test this hypothesis and fully understand the role of RFRP-3 in the seasonal control of reproduction.

Interestingly, the RFRP peptides were shown to have a modulatory action on feeding behavior (Bechtold and Luckman, 2007; Johnson et al., 2007; Clarke et al., 2012) thus it might be worth investigating whether the photoperiodic variation in RFRP expression might also impact on food intake and body weight regulation in seasonal species.

## RFRP-3 MODES AND SITES OF ACTION

The complex involvement of RFRP peptides in the regulation of the hypothalamic–pituitary–gonadal axis has raised a number of questions regarding the sites of action of the peptides. In various mammalian species including humans, RFRP fiber networks are found in multiple brain regions including the preoptic area, the arcuate nucleus, the lateral septum, the anterior hypothalamus, and the bed nucleus of the stria terminalis (Ukena and Tsutsui, 2001; Kriegsfeld et al., 2006; Johnson et al., 2007; Mason et al., 2010). Notably, RFRP-immunoreactive fibers make apparent contact with a subpopulation of GnRH neurons in rodents and sheep (Kriegsfeld et al., 2006; Smith et al., 2008; Poling et al., 2012; Rizwan et al., 2012; Ubuka et al., 2012) suggesting that RFRP-3 acts centrally to control the reproductive axis.

There is still uncertainty as to whether RFRP-3 also exerts a hypophysiotropic effect in mammals as reported in birds. A large body of evidence now reports the absence of fibers in the median eminence of rodents (Ukena and Tsutsui, 2001; Yano et al., 2003; Kriegsfeld et al., 2006; Rizwan et al., 2009; Smith et al., 2010; Ubuka et al., 2012). However, there are controversial data as to whether RFRP-3 acts (Kriegsfeld et al., 2006; Murakami et al., 2008; Pineda et al., 2010) or not (Anderson et al., 2009; Rizwan et al., 2009; Ubuka et al., 2012) on the rodent pituitary to regulate LH secretion. In the male hamster we reported no effect of the peptide on LH secretion when injected peripherally, nor on the basal and GnRH-stimulated production of LH from isolated pituitary glands (Ancel et al., 2012). In contrast in the sheep, RFRP fibers terminating in the median eminence have been identified and the peptide is released into the portal blood and appears to induce a marked inhibition of gonadotropin secretion (Clarke et al., 2008; Sari et al., 2009; Smith et al., 2012).

The RFRP peptides bind with high affinity to GPR147 (also known as NPFF1R) and with a lower affinity to GPR74 (also known as NPFF2R), which were first identified as neuropeptide FF receptors (Hinuma et al., 2000; Liu et al., 2001; Engstrom et al., 2003). The GPR147 receptor couples with Gα_i3_ and G_α__s_ proteins (Gouarderes et al., 2007) suggesting that GPR147 can have both inhibitory and stimulatory downstream effects on cellular activity. However, in CHO cells, activation of the receptor inhibits forskolin-stimulated cAMP accumulation (Mollereau et al., 2002).

NPFF receptors have been detected in rodent, lagomorph, and monkey brains suggesting that they are phylogenetically conserved (Gouarderes et al., 2004a). Importantly, however, remarkable variations in GPR147 and GPR74 receptor contents exist from one species to another and from one strain to another among the same species (Gouarderes et al., 2004a,b). Early studies describing the autoradiographic distribution of GPR147 in mice and rats indicated that the receptor was present throughout the hypothalamus (Gouarderes et al., 2002, 2004a,b). More recently, the use of *in situ* hybridization facilitated precise localization and made it possible to show that about 25% of GnRH neurons express *Gpr147*, but not *Gpr74*, in various rodent species (Poling et al., 2012; Rizwan et al., 2012; Ubuka et al., 2012). This is in agreement with the observation that RFRP-3 fibers are in contact with 20–40% of GnRH neurons (Rizwan et al., 2012; Ubuka et al., 2012). Furthermore, in mice expressing GnRH-green fluorescent protein-tagged neurons, RFRP-3 was found to exert a direct inhibitory effect on the firing rate of 41% of GnRH neurons, while 12% increased their firing rate, and the remainder were unaffected (Ducret et al., 2009). These observations support the hypothesis that RFRP-3 may exert its effects on reproduction directly via GnRH neurons. However, the peptide may also act indirectly, via upstream regulators of GnRH. This hypothesis is supported by data in rats indicating that RFRP-3 fibers are in contact with kisspeptin neurons, a subpopulation (20%) of which expresses the *Gpr147* gene (Rizwan et al., 2012).

In the Syrian hamster, RFRP-ir fibers project throughout much of the brain, including into the preoptic area and the arcuate nucleus (Kriegsfeld et al., 2006). We demonstrated that central injection of RFRP-3 to Syrian hamsters induces c-Fos expression in 30% of the GnRH neurons (**Figure 2A**) suggesting that the effects observed on the reproductive axis are mediated via these neurons (Ancel et al., 2012). Whether this effect is due to a direct action of RFRP-3 on GnRH neurons or whether it is linked to an effect on upstream regulators of the reproductive axis remains to be determined. Indeed, in the same study, although c-Fos expression was not observed in kisspeptin neurons following acute administration, the continuous central administration of RFRP-3 led to an increase in *Kiss1* expression in the arcuate nucleus together with an increase of testicular activity (**Figure 3**; Ancel et al., 2012). It is therefore possible that the RFRP-3 neuronal system regulates reproductive activity by acting at two levels of the reproductive axis: the GnRH and the kisspeptin neurons. In order to answer this question, it seems essential to carry out a detailed mapping of the *gpr147* in the Syrian hamster.

Altogether, a large amount of evidence now indicates that in various mammalian species RFRP-3 regulates reproductive activity by acting via its receptor located on GnRH neurons. This is supported by results showing that RFRP-3 fibers are in contact with a subpopulation of GnRH neurons and that *Gpr147* is expressed in GnRH neurons in rodents. However, another line of evidence points to *Kiss1* neurons as possible intermediates between RFRP peptides and the regulation of the reproductive function. Indeed, in rats RFRP-3 fibers are in contact with kisspeptin neurons which express *Gpr147* and in Syrian hamsters the reactivation of the reproductive function following continuous RFRP-3 administration goes alongside with an increase in *Kiss1* expression. Future studies using Kiss1R and GnRHR antagonists could help to understand the role of each one of these neuronal populations in mediating the effects of RFRP peptides on the reproductive axis.

## RFRP-3 AND KISSPEPTIN ACT IN CONCERT TO SYNCHRONIZE RODENT REPRODUCTION WITH THE SEASONS

There is strong evidence that RFRP neurons are regulated by photoperiod/melatonin to adapt reproductive activity to the seasons. A few years ago, the same supposition was made for kisspeptin, another member of the large RFamide family of peptides. In 2003, milestone studies reported that loss-of-function mutations of the kisspeptin receptor (KiSS1R/GPR54) in humans and rodents (de Roux et al., 2003; Seminara et al., 2003) prevented pubertal development and caused infertility, leading to a large number of studies aiming at investigating the role of kisspeptins in Vertebrate reproduction (Pinilla et al., 2012 for review). The *Kiss1* gene is mainly expressed in the arcuate nucleus and the anteroventro-periventricular nucleus of the hypothalamus, and kisspeptin neurons project specifically onto the GnRH cell bodies in the preoptic area and nerve terminals in the median eminence. In all mammalian species studied to date, kisspeptin appears as a powerful stimulator of the gonadotropic axis, acting primarily on GnRH neurons.

In the male Syrian hamster, we demonstrated that kisspeptin expression in the arcuate nucleus is down-regulated by melatonin in SD conditions, despite a negative feedback effect of testosterone on these neurons (Revel et al., 2006b). In the anteroventro-periventricular nucleus, kisspeptin expression is also decrease in SD conditions, but as a result of the absence of the positive feedback of testosterone consecutive to testicular regression (Ansel et al., 2010). Importantly, we demonstrated that chronic infusion of kisspeptin in SD-adapted sexually inactive male hamsters rescues reproductive activity to levels comparable to animals kept in photo-stimulatory LD conditions (Revel et al., 2006b; Ansel et al., 2011; Simonneaux et al., 2012). In the Siberian hamster as well, kisspeptin expression displays photoperiod variations and the peptide stimulates LH release (Greives et al., 2008a,b). Interestingly in the sheep, kisspeptin expression is also regulated by photoperiod, with a higher level of expression in SD conditions when animals are sexually active, and kisspeptin infusion in LD-adapted anestrous ewes induces ovulation in a majority of treated animals (Franceschini et al., 2006; Caraty et al., 2007; Smith et al., 2007, 2009). These observations indicate that kisspeptin expression, like RFRP, is regulated by photoperiod in seasonal species but, unlike RFRP, the direction of the regulation is different according to whether animals are LD- or SD-breeders.

To make things more complicated, other parameters also influenced by seasons regulate kisspeptin expression. This is particularly true for sex steroids which inhibit kisspeptin expression in the arcuate nucleus and increase it in the anteroventro-periventricular nucleus in various species including the Syrian (Revel et al., 2006b; Ansel et al., 2010) and the Siberian (Mason et al., 2007) hamsters. Additionally, metabolic factors that are also under the influence of seasonal changes were shown to impact on kisspeptin expression (Castellano et al., 2010). Therefore, although kisspeptin has been identified as an essential component of the photoperiodic regulation of reproductive activity in seasonal breeders (Revel et al., 2007; Simonneaux et al., 2009, 2012 for reviews) recent observations indicate that kisspeptin neurons are not the primary target of melatonin action but are controlled upstream by seasonally regulated intermediates.

Our current findings in the Syrian, Siberian, and European hamsters (Revel et al., 2008; **Figure 1**), in the jerboa (Janati et al., 2012), and other reports in the sheep (Dardente et al., 2008; Smith et al., 2008) suggest that RFRP-3 expression undergoes a conserved down-regulation by the SD melatonin signal irrespective of the reproductive response to seasons. In contrast, kisspeptin expression is increased when animals become sexually active, irrespective of the photoperiod. On the other hand, kisspeptin is always stimulatory of reproductive activity whereas RFRP-3 displays species-specific effects, being stimulatory in the Syrian hamster and inhibitory in the sheep.

**FIGURE 4:**
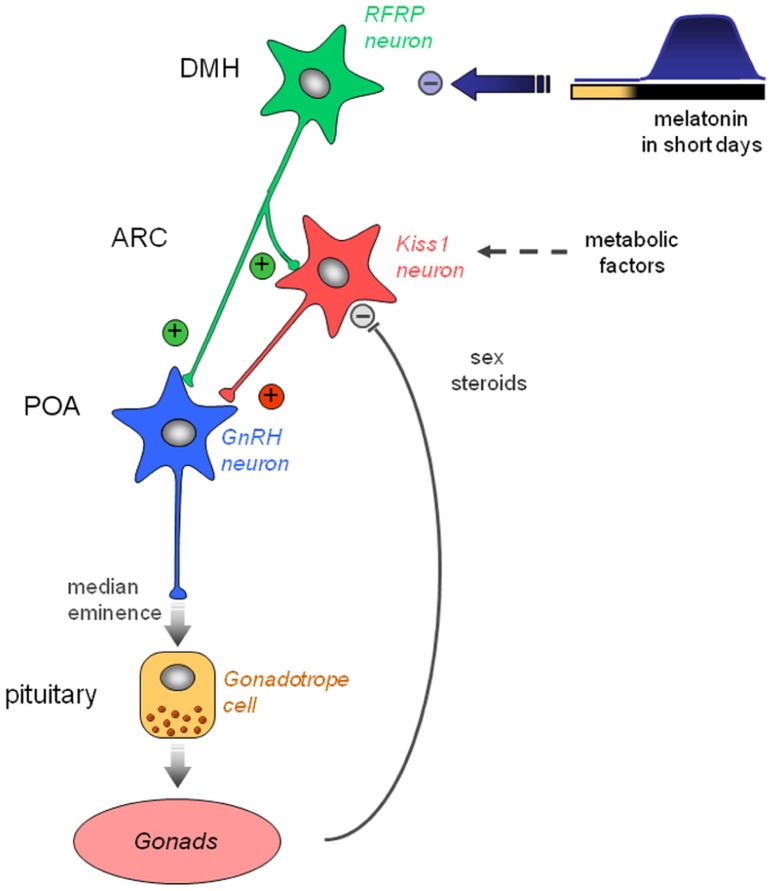
**Working model indicating how the photo-inhibitory melatonin message in short day conditions in integrated in the hypothalamus to further regulate the gonadotropic axis of the male Syrian hamster.** In long day conditions, RFRP-3 activates GnRH neuron activity directly in the preoptic area (POA) and/or indirectly via the kisspeptinergic neurons in the arcuate nucleus (ARC). In short day conditions, the large production of melatonin inhibits RFRP expression in the dorsomedial hypothalamus, which in turn decreases gonadotropic activity. *Kiss1* expression in the ARC is reduced in short day conditions, but it is also inhibited by the testosterone feedback and may be regulated by metabolic factors.

These observations have led us to propose a working model for the seasonal control of reproduction in rodents (**Figure 4**). We propose that in LD conditions, RFRP-3 would activate GnRH neuronal activity directly and/or indirectly via the kisspeptinergic neurons. The former pathway is supported by the report of RFRP-3 fibers apposed to subpopulations of GnRH neurons (Kriegsfeld et al., 2006) whereas the latter is supported both by a report in rats indicating that RFRP-3 fibers are in contact with subpopulations of kisspeptin neurons (Rizwan et al., 2012) and by our observation that chronic central infusion of RFRP-3 increases arcuate *Kiss1* expression together with a reactivation of testicular function in the Syrian hamster (Ancel et al., 2012). In SD conditions, the melatoninergic signal would be primarily integrated by the hypothalamic RFRP neurons leading to a decreased expression of the peptide, but whether melatonin acts directly or not on RFRP neurons has yet to be determined. Current studies do not support a major feedback effect of sex steroids on RFRP neurons. In contrast, kisspeptinergic neurons integrate other factors related to the sexual and metabolic status of the animal in order to finely tune reproductive activity with the seasons. From studies in sheep, a SD-breeder, it appears that an increased production of RFRP-3 in LD conditions would inhibit reproductive activity by acting directly on the GnRH neurons and/or on the pituitary (Smith et al., 2008; Sari et al., 2009).

## CONCLUSION

In regard to the seasonal regulation of reproductive activity, recent data have shed light on the involvement of two hypothalamic peptides of the RFamide family, RFRP-3 and kisspeptin. Clearly both peptides are regulated by the photoperiodic melatoninergic signal but may also be sensitive to other seasonally regulated factors. Our current hypothesis is that RFRP expression undergoes a conserved inhibition in short photoperiod but the peptide may be stimulatory or inhibitory according to the reproductive physiology of the species; on the other hand, kisspeptin has a conserved stimulatory action on the gonadotropic axis but its seasonal regulation shows species differences. Whether RFRP-3 and kisspeptin act on each other’s expression or independently to regulate reproductive activity has yet to be clarified in different species. In the years to come, we believe that thorough comparative analyses on the effects and sites of action of both peptides between LD- and SD-breeders should help to resolve the yet unanswered question of why the same photoperiodic cue induces opposite behavioral responses.

## Conflict of Interest Statement

The authors declare that the research was conducted in the absence of any commercial or financial relationships that could be construed as a potential conflict of interest
